# Rapid Peptides Generator: fast and efficient *in silico* protein digestion

**DOI:** 10.1093/nargab/lqz004

**Published:** 2019-10-05

**Authors:** Nicolas Maillet

**Affiliations:** Hub de Bioinformatique et Biostatistique - Département Biologie Computationnelle, Institut Pasteur, USR 3756 CNRS, Paris, France

## Abstract

Recent developments in mass spectrometry techniques used in proteomics and proteogenomics have led to a constantly increasing interest in proteases. These proteases are used in different mass spectrometry analyses requiring protein digestions. To perform such digestions, one or multiple proteases are used. Few software exist that predict cleavage sites of proteases and simulate *in silico* digestions. In this work, Rapid Peptides Generator (RPG), a new software developed in order to predict proteases-induced cleavage sites on sequences, is presented. RPG offers extra features and overcomes most issues of existing software in different ways. First, for each generated peptide, RPG gives its sequence, length and estimation of mass, measurements already provided by other software, as well as the peptide’s isoelectric point. Moreover, contrary to existing software that limit the option of proteases to be used to a predefined list, users of RPG are able to easily define new proteases using a simple yet powerful grammar. This feature allows users to stay up-to-date to new or more specific proteases available on the market and optimizes time and effort before the actual mass spectrometry experiment. RPG is freely available through the well established package management system ‘pip’ and follows the standards for software development.

## INTRODUCTION

Proteases, also known as proteolytic enzymes, have been studied for more than 80 years (1). They are widely used in industry, medicine as well as a biological research tool, for example in protein characterization, proteomics and proteogenomics (2).

Recent developments in mass spectrometry (MS) techniques used in proteomics and proteogenomics have led to a constantly increasing interest in proteases. In ‘bottom-up’ analysis, using tandem mass spectrometry (MS/MS), the optimal peptide size range is 600–5000 Da (3) when protein sizes are usually more than 10 000 Da. Therefore, for bottom-up approaches, protein digestions are required. To perform such digestions, one or multiple proteases, like trypsin, pepsin or thrombin, are used. Each protease has specific cleavage sites depending on solvent accessibility, pH, temperature etc. The use of different proteases individually or in combination creates a unique set of peptides. Performing multiple digestions can increase overall confidence in protein identification, if cleaving sites are different. It is not straightforward to determine which combination of proteases will lead to a set of peptides suitable for MS/MS analysis. However, the cost of some proteases does not allow for easily trying multiple combinations in order to avoid redundancy of cleaving sites. To this end, few software that predict cleavage sites of proteases in protein sequences have been developed. Among those, the most commonly used are PeptideCutter from ExPASy Server (4) and a module integrated in MaxQuant (5).

PeptideCutter performs a digestion using one or multiple proteases, among a total list of 38, and provides detailed results, including positions of cleavage sites, peptide sequences, lengths and masses. Despite the valuable information provided by this software, three main features are missing. First, in order to thoroughly analyze the behavior of a specific combination of proteases, it is important to try this combination on many different proteins. With PeptideCutter, the user cannot perform parallel or automatic sequential digestions of different proteins sequence and thus this procedure is time consuming and not efficient. The second drawback of this tool is how a combination of proteases is assessed. In PeptideCutter, all selected proteases are assumed to be present at the same time during digestion. It is therefore difficult to simulate distinct digestions, i.e. digestions of the same sequence using different proteases separately. This means that instead of an automatic succession of distinct digestions, one has to run the software as many times as the number of distinct digestions, multiplied by the number of sequences. Last but not least, in PeptideCutter it is not possible to input novel protease definitions, which is not adapted to the increasing number of new or more specific proteases (denoted as ‘Sequencing Grade’ or SG). Depending on the company manufacturing those SG proteases, specificity and definition can change. Hence, it is important for the user to easily adapt the software by including novel definitions of proteases. The alternative, MaxQuant, partially overcomes some of those issues. The user can input new protease definitions by specifying between which amino acids cleavages occur. Unfortunately, this definition is not sufficient to properly define some proteases. For example, definition of trypsin in MaxQuant lacks some exceptions, e.g. it is defined as cleaving after K or R, but not before P (see Table 1 for amino acid designation). However, it has been reported that although most of the times P blocks the cleavage when found after K, this is not true when K is preceded by W: a cleavage occurs after K in ‘WKP’ motif (6). Currently, it is not possible to create such rules in MaxQuant.

**Table 1.** Molecular weight of amino acid and p*K*_*a*_ values of ionizable groups in proteins

Amino acid	1-letter	Mass value*	Typical p*K*_a_**	p*K*_*a*_ from
	Abbreviation	(Da)		IPC_peptide
N-terminal	n	–	8.0	9.564
Alanine	A	71.0788	–	–
Cysteine	C	103.1388	8.3	8.297
Aspartic acid	D	115.0886	4.1	3.887
Glutamic acid	E	129.1155	4.1	4.317
Phenylalanine	F	147.1766	–	–
Glycine	G	57.0519	–	–
Histidine	H	137.1411	6.0	6.018
Isoleucine	I	113.1594	–	–
Lysine	K	128.1741	10.8	10.517
Leucine	L	113.1594	–	–
Methionine	M	131.1926	–	–
Asparagine	N	114.1038	–	–
Pyrrolysine	O	237.3018	–	–
Proline	P	97.1167	–	–
Glutamine	Q	128.1307	–	–
Arginine	R	156.1875	12.5	12.503
Serine	S	87.0782	–	–
Threonine	T	101.1051	–	–
Selenocysteine	U	150.0388	–	–
Valine	V	99.1326	–	–
Tryptophan	W	186.2132	–	–
Tyrosine	Y	163.1760	10.9	10.071
C-terminal	c	–	3.1	2.383

*Average molecular weight as defined in ExPASy web server (https://web.expasy.org/findmod/findmod_masses.html#aas)

**p*K*_*a*_ values as defined in Biochemistry (10) . Values depend on temperature, ionic strength and the microenvironment of the ionizable group.

In this paper, a novel software developed to predict proteases-induced cleavage sites on sequences is presented, Rapid Peptides Generator (RPG), overcoming most issues of existing programs. First, RPG computes an accurate estimation of the molecular weight and isoelectric point of each generated peptide. Second, RPG takes into account miscleavages and importantly, it assigns to each proteases a probability of miscleavage event. Third, RPG introduces two distinct digestion modes. In the first one, each selected protease acts independently, simulating different experiments on multiple proteins. In the second mode, all selected proteases are acting at the same time, simulating multiple proteases digestions of a protein. Finally, the main contribution of RPG is the possibility for the user to easily define new proteases and use them in the software.

RPG is freely available through the well established package management system **pip** (*pip3 install rpg*) and follows the standards for software development with continuous integration on Gitlab (https://gitlab.pasteur.fr/nmaillet/rpg) and automatic on-line documentation (https://rapid-peptide-generator.readthedocs.io).

## MATERIALS AND METHODS

RPG is a python tool that takes (multi-)fasta/fastq file of proteins as input and digests each of them. Digestion mode can be either ‘concurrent’, i.e. all proteases are present at the same time during digestion or ‘sequential’. In sequential mode, each sequence is digested by each protease, one by one. In both modes, the output information is the same as in PeptideCutter, plus an important property: an estimation of isoelectric point (pI) of each generated peptide. Shortly, the isoelectric point is the pH at which a peptide carries no net electrical charge and a good approximation can be computed on small molecules (7). The results are outputted in multi-fasta, CSV or TSV file.

At the moment, 42 proteases and chemical compounds are included in RPG. The user can easily design new proteases, using a simple yet powerful grammar. This grammar enables the user to design complex proteases like trypsin or thrombin, including many exceptions and different cleavage sites. User-defined proteases are then interpreted by RPG and included in the local installation of the software.

In the rest of the text, nomenclature of Schechter and Berger (8) is used. Amino acids before the cleavage site are designated as P1, P2, P3, etc. in the N-terminal direction, and as P1’, P2’, P3’, etc. in the C-terminal direction. For example, with cleavage site represented by an arrow (↓), amino acids are designated as:

...P3-P2-P1-↓-P1’-P2’-P3’...

Note that in RPG, this nomenclature is represented as:

...(P3)(P2)(P1)(,)(P1’)(P2’)(P3’)...

### Definition of cleaving site: the RPG *rule* object

The main structure of RPG is a recursive object named *rule*. A *rule* is defined by an amino acid of interest (amino_acid in all following examples), at a relative position (index), and by a Boolean indicating if the program should cleave (cleavage) before or after (position) this specific amino acid. For example, the *rule*rule_a = (a, 0, True, Before) indicates to cleave before the current position (0), when an alanine (a) is encountered. The *rule*rule_b = (e, 0, True, After) will cleave just after a glutamate acid (e).

The *rule* also contains a list of *rule* objects (sub-rules), hence its recursivity. This list represents exceptions to the main *rule* they are linked to. In the previous example, i.e. rule_b = (e, 0, True, After), one can add an exception that specifies not to cleave if the glutamic acid is preceded by another glutamic acid. To do so, the *rule*sub_rule_b = (e, -1, False, None) must be added to the *sub-rules* list of rule_b. It should be noted that for the *sub-rules*, the cleaving indication (before or after) is not relevant and the position is relative to the position of the amino acid in the main *rule*. The sub_rule_b indicates to look at the *n* − 1 position: if an e is found there, no cleavage occurs after the e at the current position. Finally, the rule_b defines a protease that cleaves after an e if this e is not directly preceded by another e. This protease reproduces the behavior of staphylococcal-peptidase-I that preferentially cleaves after e (P1). It will not cleave after e in P1 preceded by e in P2, but it cleaves after e in P1 followed by e in P1’ (e↓e). The sequence aeert will be cleaved by staphylococcal-peptidase-I only after the first e, resulting in two peptides: ae and ert. A way of representing staphylococcal-peptidase-I with RPG *rule* objects is show on Rule 1.


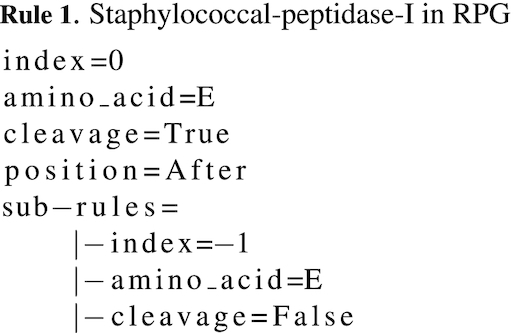


This sub-rules system can also work the other way: cleaving if a specific amino acid is present after/before another specific one. For example, hydroxylamine preferentially cleaves after n (P1) followed by g in P1’ (n↓g). To define this protease (see Rule 2), the main *rule* will specify to ‘not’ cleave after n while the sub-rule will specify to actually cleave if g is following:


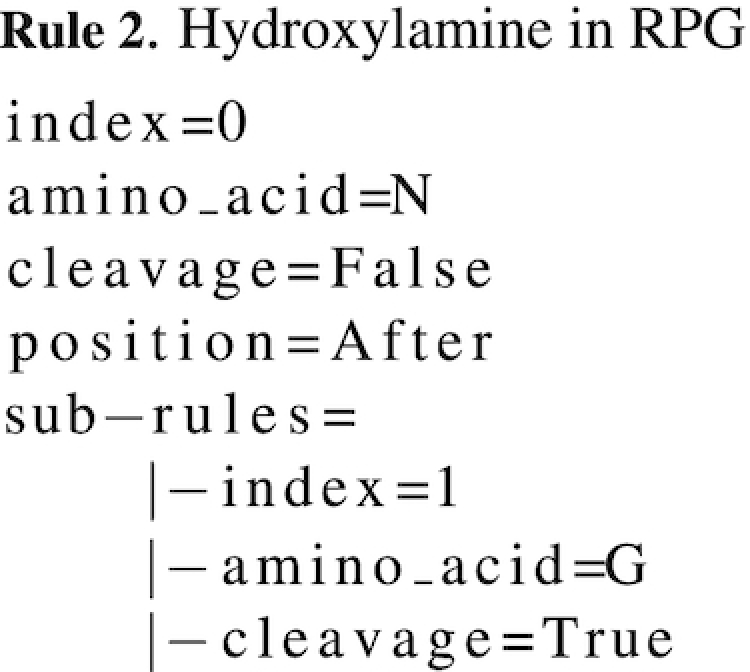


Note that sub-rules are *rules*, hence the possibility to assign sub-rules to sub-rules. For example, thrombin SG as defined in RPG preferentially cleaves after r (P1) preceded by p in P2, v in P3 and l in P4, and followed by g in P1’ and s in P2’. As shown in Rule 3, only the deepest sub-rule will contain the information to cleave as all upper amino acids are required. This means that thrombin SG will only cleave after r on the specific sequence lvpr↓gs: if one amino acid is missing, no cleavage occurs.


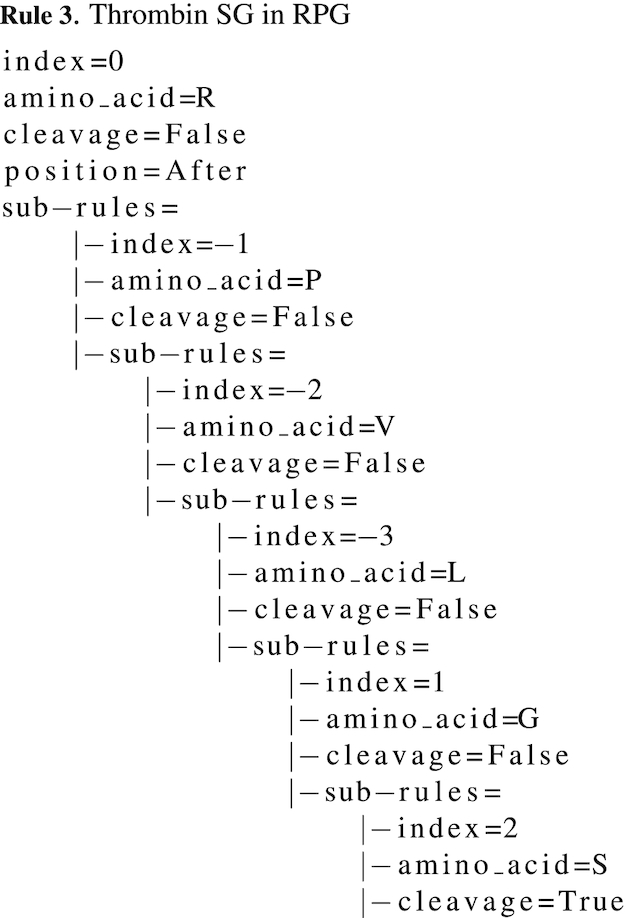


In the previous examples, a protease is defined in RPG as a single rule, with potential sub-rules. More generally, a protease is defined by a list of *rule*. To fully describe the chymotrypsin (high specificity) in RPG, three rules are needed: chymotrypsin (high specificity) preferentially cleaves after f, y or w (P1) if those amino acids are not followed by p in P1’. Moreover, it will not cleave after w followed by m in P1’. Thus, three *rules* are defined, one for f, one for y and one for w. Those three *rules* have an identical sub-rule, defining the exception when p is following their amino acid. The above mentioned *rule* for w also contains a second sub-rule, preventing the cleavage when an m is following it. Rule 4 presents the *rules* in RPG for chymotrypsin (high specificity).


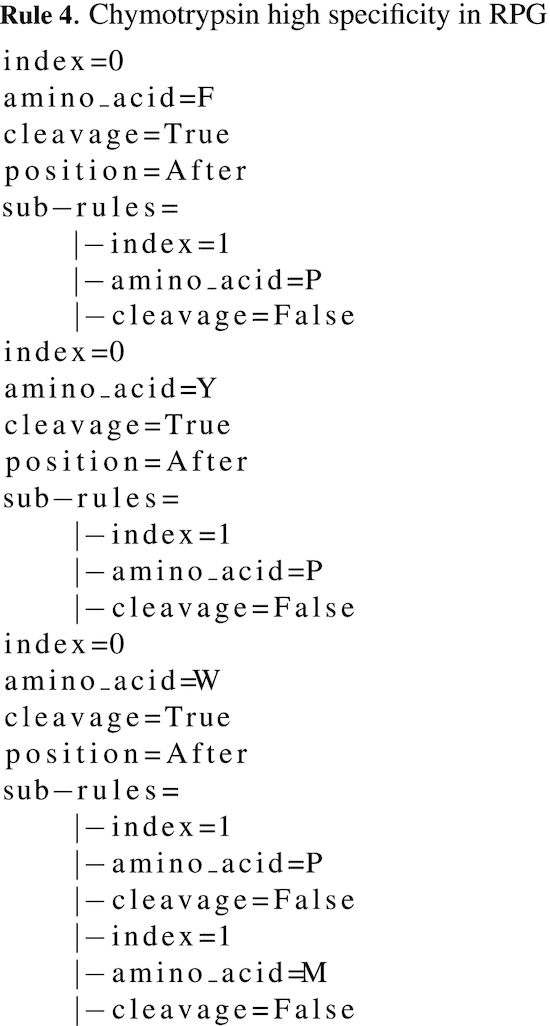


No database is needed to store proteases as each protease is just composed of Python executable code. This code is used by RPG engine to deduce where cleavages occur on a sequence.

### Cleaving sequences: RPG engine

The second key part of RPG is the engine interpreting proteases defined by *rules*. This main function takes a sequence and a protease as input. The sequence is then processed amino acid by amino acid. For each position, all main *rules* of the protease are analyzed to check if the current amino acid corresponds to one of them. Note that sub-rules are not taken into account at this point: the program just verifies if the current amino acid is involved by a potential cleavage.

When an amino acid corresponds to one of the main *rules*, the sub-rules are recursively tested to define if a cleavage occurs. If a cleavage occurs, the peptide corresponding to the left part of the original sequence until the cleavage position is generated and the current position is kept in memory. Note that the original sequence is not truncated, as potential future cleavage points may require information from previous amino acids (for example, staphylococcal-peptidase-I, see Rule 1). When another cleavage occurs, the new peptide will be generated from the previous stored position until the current cleavage position. At the end of the sequence, the last peptide is generated from the last stored position until the end of the sequence.

When a peptide is generated, several estimations are computed as explained in the following section.

### Molecular mass and isoelectric point estimations

For each generated peptide, three measurements are computed: the length of the peptide, its estimated molecular weight and its estimated isoelectric point (pI). The length is simply the number of amino acids composing the peptide.

The molecular mass of a peptide is less direct to compute. Since proteolysis requires a molecule of water, after a cleavage the chemical structure of the resulting peptide is slightly modified: a hydrogen is added at the N-terminus and a hydroxyl group is added at the C-terminus. The mass of two hydrogens and one oxygen must then be added to the total mass of the peptide. The resulting mass of a peptide is calculated by adding up the average isotopic masses of each amino acid present in the peptide and the average isotopic mass of one water molecule. Note that in RPG, no post-translational or digestion-induced modifications are included in the results.

The pI of a peptide is the pH at which this peptide is electrically neutral. The pI is of great relevance in biochemistry, especially in liquid chromatography mass spectrometry. As its net charge is directly related to the pH of the solution, the isoelectric point can be computed by solving the following rearranged Henderson–Hasselbalch equations (9), which calculate the charge of a peptide at a certain pH:

$$\begin{eqnarray*} {\rm pI} = \left\lbrace \begin{array}{rcr}\sum \limits _{i=1}^{n}\frac{-1}{1+10^{pK_a-pH}} \text{, for negatively charged amino acids}\\ \sum \limits _{i=1}^{n}\frac{1}{1+10^{pH-pK_a}} \text{, for positively charged amino acids}\\ \end{array} \right. \end{eqnarray*}$$

where p*K*_*a*_ is the acid dissociation constant of the given charged amino acid (see Table 1) and *n* the number of amino acids.

Note that in those equations, only the pH is variable while the p*K*_*a*_ values are fixed. Given fixed p*K*_*a*_ values, the net charge of a peptide at a given pH is simply the sum of positive and negative charges of all amino acids composing it.

Typical p*K*_*a*_ values of ionizable groups are well-defined in literature (10). A recent paper (IPC – Isoelectric Point Calculator (7)) showed that p*K*_*a*_ values can be more accurately defined for different classes of macromolecules.

As mentioned earlier, no post-translational or digestion-induced modifications are included in RPG, leading to a result almost certainly different from the exact pI (9). Nevertheless, given appropriate p*K*_*a*_ values, the calculated approximation is ±0.5 of the exact pI of proteins and it is even better for short peptides (7). In RPG, p*K*_*a*_ values from IPC_peptides are used by default, with the possibility for the user to select more typical p*K*_*a*_ values. The pI is computed using binary search. The computation starts by solving Henderson–Hasselbalch equations for the mean pH 7. If the result is <0 (respectively, >0), the pH corresponding to this pI is most certainly <7, so between pH 0 and 7 (>7, so between pH 7 and 14). Henderson–Hasselbalch equations are then solved using the mean pH of the resulting interval: pH 3.5 (pH 10.5). This computation will continue until a suitable pH value is found, with an accuracy of 0.01.

### New protease creation

One of the main features of RPG is the possibility for users to easily define new proteases. As explained in section **Definition of cleaving site**, proteases are defined in RPG as instructions of Python code. To simplify the description of new proteases without having to write code, a new formal grammar has been created. This grammar, as any formal grammar, is composed of an alphabet, a syntax and a set of production rules to form valid strings, according to the grammar definition.

To stay as close as possible to the nomenclature of Schechter and Berger, the grammar of RPG introduces only minor modifications, allowing it to be formal and easier to use. For example, hydroxylamine, which cleaves between n and g (n↓g), and represented as n(P1) g(P1’) using the nomenclature of Schechter and Berger, is defined as (n,)(g) in RPG’s grammar. A protease definition is composed of one or several rules, expressed using the grammar.

#### Basic core of the grammar

Each inputed rule consists of sequences of involved amino acids. The first modification is the formal definition of the position of the cleavage site. As a matter of fact, even if a rule is composed of only one amino acid, there is still possibility to cleave before or after this amino acid. Hence, the formalism of RPG’s grammar necessitates a comma ‘,’ to indicate the cleavage site. For example, hydroxylamine will be defined as n,g, indicating that cleavage must occur between n(P1) and g(P1’). Note that each rule must contain only one comma, i.e. one cleavage site: if a protease cleaves at different positions, several rules must be defined. This beginning of formalism induces the concept of and between amino acid: n,g represent a cleavage if n**and**g are both present, in this specific order.

In order to facilitate the definition of more complex proteases, two adjustments were included.

#### The or keyword and parenthesis

With the basic grammar defined above, one can input rules corresponding to Bromelain that preferentially cleaves after k, a or y (P1). To do so, one must input three rules: k,, a, then y,. To simplify this procedure, the or keyword was introduced. Bromelain can then be defined with a single rule: k, or a, or y,.

There are, however, cases where this straightforward and simple rule can lead to ambiguous definitions: does the rule ac, or d, define a cleavage occurring after ac or d, or occurring after ac or ad? To resolve this ambiguity and simplify the reading, parentheses were introduced. A parenthesis system, which is everything between an opening parenthesis and a closing parenthesis, represents a position, like P1, P2, P1’, etc. This constrains the grammar and thus the previous example will not be valid anymore. It will either be the rule (a)(c or d,), simulating a protease cleaving after c or d (P1) when a is in P2, or the two rules (a)(c,) and (d,), cleaving after c (P1) when a is in P2 or always after d (P1). With this parentheses system, Bromelain definition becomes (k or a or y,). Note that there is now a single comma, indicating whereas k, a and y are in P1 or in P1’ and that the or keyword is evaluated before the comma.

#### Two commas

With the parentheses system only one comma is required per rule; a comma is where the cleavage occurs, before or after the involved position. For an easier usage, a rule can contain two commas as long as they are in the same parentheses system; this corresponds to a cleavage occurring before and after a specific amino acid. For example pepsin, used when pH is >2, preferentially cleaves around f, l, w or y (P1 or P1’). This can be defined in RPG as a set of two rules: (,f or l or w or y) and (f or l or w or y,). Using two commas, this becomes the single rule: (,f or l or w or y,). Note that in reality pepsin cleavage rules are more complicated and contain many exceptions in its corresponding RPG full definition.

#### Exceptions

The last important part of protease definition in RPG is the notion of exceptions. An exception is a rule modifying the behavior of a normal cleavage. For example, staphylococcal-peptidase-I cleaves after e (P1) when there is no e in P2. The main rule is to cleave after e and the exception is to not cleave if another e precedes it. An exception in RPG follows the same grammar as a classical rule. The only difference is RPG's interpretation: an exception is a sub-rule of a normal rule, i.e. it will be added in the list of rules of the involved main rules (see section **Definition of cleaving site**). This requires for the main rule to be already defined in the current protease definition, as every exception must be linked to a main rule. Note that a normal rule can already contain exceptions; for example in the hydroxylamine definition ((n,)(g)), g is an exception added to the sub-rule list of the main rule n (see Rule 2). In hydroxylamine, this mechanism is transparent for the user, as no proper exceptions are defined by her/him. The exception mechanism described here allows the user to manually define an exception that is impossible to describe using normal rules.

### Miscleavage

It is possible that a protease does not cleave at a given position even if all requirements are fulfilled. This event is called miscleavage and can have biological, chemical or physical origins. Despite the existence of some empirical miscleavage rules, there is an uncertainty concerning which sites will be cleaved or not (11). Moreover, depending on the protease, the experimental conditions and exposure time, the miscleavage events probability may vary from very small to high. In RPG, a simple method is used in order to take into account these miscleavage events: when running RPG, one can assign a miscleavage probability to each selected protease, expressed as a percentage. Then, every time RPG finds a cleavage position, a random number that reflects the miscleavage probability is generated: if the generated number is below the percentage probability, no cleavage occurs. For example, for a specific and aggressive protease, such as trypsin in optimal conditions, the number of miscleavages is low: thus a value of 0 to 1% is appropriate. For a non-specific protease like pepsin, used at a low concentration and exposure time, a value of 20% can be considered. While this method does not take into account the biological, chemical or physical origins it does incorporate the concept of miscleavage, which is crucial. Indeed, without it, RPG delivers a perfect cleaving situation, that rarely occurs in reality.

## RESULTS

In the following, RPG is compared to PeptideCutter, the current gold standard for *in silico* digestion. RPG and PeptideCutter are compared in terms of results and execution time. RPG is then applied on two protein families with three different sets of proteases. Results exhibit how the choice of proteases greatly conditions the nature of the generated peptides.

### Comparison between RPG and PeptideCutter

The 38 proteases available in PeptideCutter are also available in RPG and are compared in this section. As PeptideCutter can only work on one protein at a time, one of the largest proteins has been chosen to run the following benchmark: the Human muscle protein Titin (UniProtKB id: A0A0A0MTS7), which consists of 35 991 amino acids (12).

RPG's execution time was obtained using the time command of the Unix system. PeptideCutter's execution time is the effective time needed for the web server to fully output the result page in the browser. In both cases, execution time includes the computation time and the time to output results. The mean execution time of RPG for digesting Titin with one protease is 0.467 s (see Table 2). For PeptideCutter the mean time is computed without caspases 3 to 10 as the computation for those proteases was long and aborted after 30s. PeptideCutter’s mean time is 3.446 s and execution time of RPG is, on average, 7.379 times faster than PeptideCutter.

**Table 2.** Comparison of PeptideCutter and RPG in terms of execution time, number of generated peptides and results

Protease	Execution time (s)		Number of peptides		Identical
Name	RPG	PepC	RPG	PepC	Results
Arg-C	0.415	2.86	1665	1665	True
Asp-N	0.463	3.99	2261	1739	False
Asp-N-pepc*	0.421	3.99	1739	1739	True
Asp-N-Glu-N	0.594	4.43	5175	5175	True
BNPS-Skatole	0.371	2.59	470	470	True
Caspase1	0.346	3.8	22	22	True
Caspase2	0.364	2.86	2	2	True
Caspase3	0.389	>30	1	–	–
Caspase4	0.372	>30	1	–	–
Caspase5	0.356	>30	1	–	–
Caspase6	0.362	>30	1	–	–
Caspase7	0.365	>30	1	–	–
Caspase8	0.357	>30	1	–	–
Caspase9	0.406	>30	1	–	–
Caspase10	0.409	>30	1	–	–
Chymotrypsin-high	0.488	3.23	2334	2334	True
Chymotrypsin-low	0.661	4.73	5188	5188	True
Clostripain	0.414	3.35	1665	1665	True
CNBr	0.360	2.42	409	409	True
Enterokinase	0.338	3.16	1	4	False
Enterokinase-pepc*	0.348	3.16	4	4	True
Factor-Xa	0.344	2.53	8	8	True
Formic-acid	0.416	2.99	1739	1739	True
Glutamyl	0.495	3.37	3437	3437	True
GranzymeB	0.349	2.28	2	2	True
Hydroxylamine	0.345	2.88	50	50	True
Iodosobenzoic-acid	0.362	2.39	470	470	True
Lys-C	0.493	3.41	3187	3187	True
Lys-N	0.491	3.47	3187	3187	True
NTCB	0.363	2.53	523	523	True
Neutrophil-elastase	0.612	3.97	5645	5645	True
Pepsin-pH1.3	0.603	3.99	4399	4399	True
Pepsin-pHg2	0.771	4.45	6482	6482	True
Proline	0.362	2.61	408	408	True
Proteinase-K	1.298	6.96	18393	18393	True
Staphylococcal-p-I	0.482	3.35	3092	3092	True
Thermolysin	0.831	4.48	8835	8835	True
Thrombin	0.346	2.54	4	4	True
TEV	0.344	2.96	3	7	False
TEV-pepc*	0.347	2.96	7	7	True
Trypsin	0.566	4.27	4425	4425	True
Arg-C-LysN-BNPS	0.803	4.19	5163	5163	True

*Proteases locally defined in RPG and not available in default installation.

Contrary to PeptideCutter, RPG can run sequentially multiple proteases, where PeptideCutter performs as if all selected proteases were cleaving at the same time. This mode of RPG allows to calculate all the cleaving operations in a single run. Simulating the cleaving site and generating the peptides for the 38 tested proteases of Table 2 took 6.141 s with RPG.

Except for three proteases, all results are strictly identical, with exactly the same mass for each generated peptide (precision of three numbers induced by PeptideCutter) and the same number and content of peptides. The three proteases with different results are Asp-N endopeptidase, enterokinase and Tobacco etch virus protease (TEV). The definition of Asp-N endopeptidase as established in PeptideCutter cleaves specifically before aspartic acid, whereas the definition in RPG is less restrictive, cleaving before aspartic acid or cysteine, as defined in several publications (10,13,14).

In theory, enterokinase and TEV have long cleavage definitions, i.e. a succession of five amino acids for enterokinase ((d or e)(d or e)(d or e)(d or e)(k,)) and seven for TEV ((e)()()(y)()(q,)(g or s)). As mentioned in the documentation of PeptideCutter, it does not take into account positions P5 and later, leading to implemented definitions shorter than theoretical ones. RPG, on the contrary, implements the full definitions.

Because RPG gives the opportunity to easily add new proteases, those three proteases were added in the local RPG installation and are represented in Table 2 as Asp-N-pepc, enterokinase-pepc and TEV-pepc. Those results are strictly identical to peptidCutter results.

The last entry of Table 2, namely Arg-C-LysN-BNPS, is the digestion of Titin by three proteases: Arg-C, Lys-N and BNPS-Skatole. PeptideCutter performs this operation as if all proteases cleave together simultaneously. This corresponds to RPG's concurrent mode. Results are identical for a total of 5163 peptides.

### Real case studies

RPG was used on two protein families: Actins and Globins. The Representative Proteome (RP15) of Actins and Globins were retrieved from Pfam (15) (id: PF00022 and PF00042). Actin dataset is composed of 5704 sequences for a total of 1 545 777 residues. Globin dataset is composed of 1678 sequences for a total of 161 696 residues.

On these datasets, RPG was used with two different sets of proteases. The results were then analyzed to determine the proportion of generated peptides for each set having a suitable molecular weight for tandem mass spectrometry analysis (600–5000 Da).

**Figure 1.**
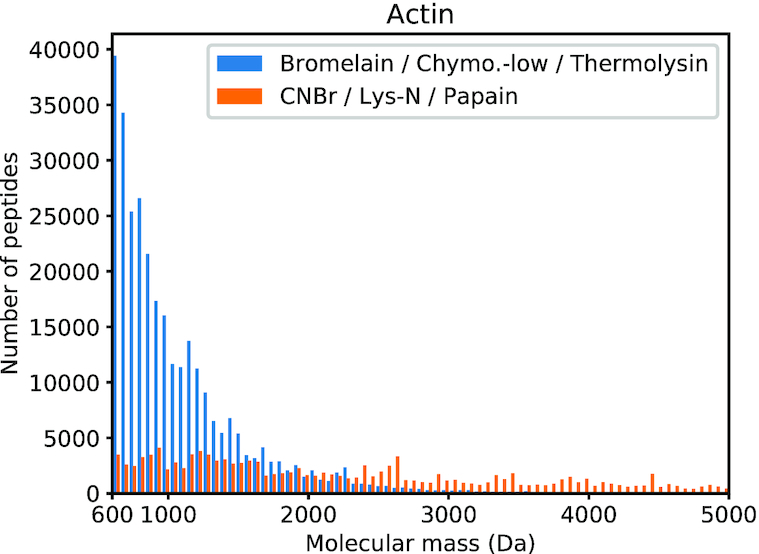
Number of generated peptides by proteases set A (Bromelain, Chymotrypsin-low and Thermolysin) and proteases set B (CNBr, Lys-N and Papain) in the optimal peptide size range (600–5000 Da) for the Actin protein family. Set A is more appropriate to obtain peptides in the analyzed range.

**Figure 2.**
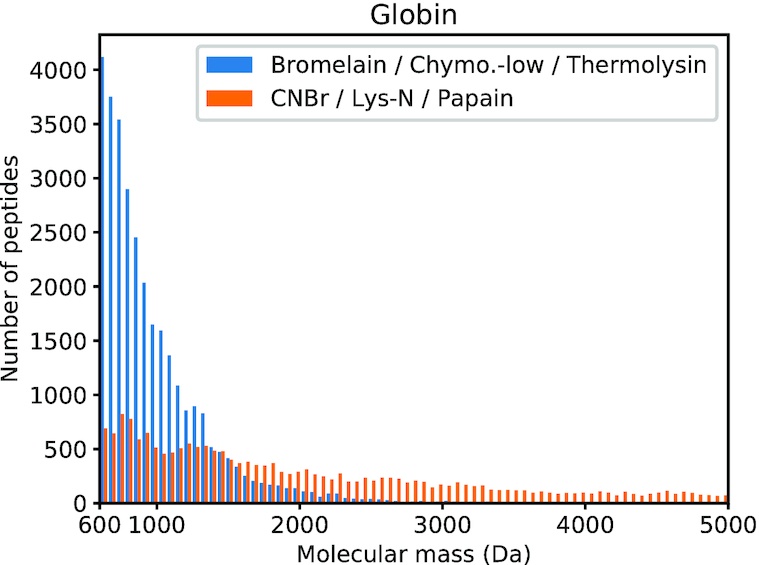
Number of generated peptides by proteases set A (Bromelain, Chymotrypsin-low and Thermolysin) and proteases set B (CNBr, Lys-N and Papain) in the optimal peptide size range (600–5000 Da) for the Globin protein family. Set B is more appropriate to obtain peptides in the analyzed range.

The first set (set A, in blue on Figures 1 and 2) is composed of the three proteases Bromelain, Chymotrypsin-low and Thermolysin, used sequentially. The second set (set B, in orange on Figures 1 and 2) is composed of the three proteases CNBr, Lys-N and Papain, used sequentially.

#### Actin family

Set A generates 1 037 886 peptides on Actin RP15 (total of 4 637 331 residues: three proteases, three times each sequence). Among these peptides, 302 694 have a molecular mass between 600 and 5000 Da (29.16%), see Figure 1. These peptides are composed of 2 846 676 residues (61.39% of the total number of residues).

Set B generates 187 796 peptides (total of 4 637 331 residues). Among these peptides, 125 419 have a molecular mass between 600 and 5000 Da (66.78%). These peptides are composed of 2 422 798 residues (52.25% of the total number of residues).

Interestingly, set A generates many short peptides: 70.82% have a molecular weight inferior to 600 Da. Nevertheless, these peptides contain only 38.42% of the total number of residues and the vast majority of the generated residues are inside the desired molecular weight range. On the contrary, set B generates bigger peptides: 15.32% are actually too heavy, accounting for 45.78% of the total number of residues. Even if the majority of peptides generated by set B lies in the desired range, less residues are actually involved as compared to set A, with a difference of more than 400 000 residues. Compared to set B, the combination of Bromelain, Chymotrypsin-low and Thermolysin is more appropriate to properly digest proteins of the Actin family, in order to perform MS/MS analysis.

#### Globin family

Set A generates 129 616 peptides on Globin RP15 (total of 485 088 residues). Among these peptides, 30 990 have a molecular mass between 600 and 5000 Da (23.91%), see Figure 2. These peptides are composed of 261 524 residues (53.91% of the total number of residues).

Set B generates 27 725 peptides (total of 485 088 residues). Among these peptides, 19 185 have a molecular mass between 600 and 5000 Da (69.2%). These peptides are composed of 325 476 residues (67.1% of the total number of residues).

Similarly to the case of Actins, set A generates many short peptides (76.09%). Set B also leads to bigger peptides, but for the Globins the big majority of generated peptides (69.2%) are in the desired range and correspond to 67.1% of the total number of residues. Compared to set A, the combination of CNBr, Lys-N and Papain is more appropriate to properly digest proteins of the Globin family, in order to perform MS/MS analysis.

#### Other combination

Another combination of proteases (Chymotrypsin-high/Ficin/Trypsin) leads to better results for both Actins and Globins. For Actins, it leads to 275 647 desired peptides among the 434 452 generated (63.45%), which represent 4 000 384 of the 4 637 331 total residues (86.26%). For Globins, 30 740 peptides among the 51 893 generated (63.45%) are desired, representing 407 299 of the 485 088 total residues (83.96%).

## DISCUSSION

The recent increase of interest in proteases, linked to advancements in mass spectrometry techniques, can be enhanced by software tools. In ‘bottom-up’ analysis, protein digestions are required. *In silico* digestions are a quick and inexpensive way of selecting proteases for a particular class of proteins. To be efficient, *in silico* digestion must be realistic, accurate and easily adapted to user’s proteases. In the following, contributions of RPG are discussed, in terms of functionalities and results.

### Digestion modes

RPG introduces two distinct digestion modes. The first one (sequential) simulates distinct digestions with different proteases. Each selected protease cleaves independently and produces distinct results. This mechanism is, in terms of results, identical to running *N* times RPG, *N* being the number of different proteases. This first digestion mode simplifies operations and speeds up the process of obtaining results for *N* distinct proteases. The second digestion mode (concurrent) also takes several proteases but simulates the behavior of those proteases as if they were all digesting at the same time, for an infinite time. The particularity of this digestion mode is the ability for one protease to access a cleavage site that it normally not available. This happens when a protease cleaves a protein and the resulting peptides are then cleaved by another protease that would not have been able to cleave the original protein. For example, a protease cleaving before p (P1’), if this amino acid is not followed by k in P2’, will not be able to cleave a pattern xxpkxx where x represents any amino acid. In concurrent mode, the user can mix this protease with Lys-N, which cleaves before k (P1’). Lys-N will then cleave the pattern, leading to two peptides: xxp and kxx. The first protease can then access the p and will cleave the first peptide, leading to a global result of three peptides: xx, p and kxx. While this example is trivial, some combinations of proteases can lead to behaviors that are difficult to assess without a proper simulation. The concurrent mode of RPG is useful to analyze these situations.

### Accuracy of molecular weight and isoelectric point

Molecular weight and isoelectric point are two important measures to assess the behavior of a mass spectrometry pipeline. RPG gives accurate estimations of these two measures.

Molecular weight is critical for mass spectrometry analysis, since the composition of studied proteins is estimated based on this weight. Hence, it is important for *in silico* digestion to perform a good approximation of the peptide’s weight. RPG delivers a precision of four digits. Note that the computation of mass is based on the average masses of the amino acids.

Similarly, the computation of isoelectric point is of great importance. Before the mass spectrometry analysis, peptides are separated into fractions, using for example chromatography. This separation is usually based on isoelectric point and fractions allow to reduce mass spectrometry analysis complexity. An accurate estimation of pI is then necessary to properly simulate *in silico* digestion for purposes of mass spectrometry analysis. RPG delivers a good estimation of pI, based on previous works.

### Designing new proteases with RPG grammar

The grammar of RPG gives the user the possibility to use the and operator (see **Basic core of the grammar**), the or operator (see **The****or****keyword and parenthesis**) and the not operator (see **Exceptions**). The combination of these three operators offers the possibility to the user to easily define any kind of complex protease.

One complex example is trypsin. According to PeptideCutter (4), trypsin preferentially cleaves at k and r in position P1. This is expressed in RPG grammar as (k or r,). p usually blocks the action when found in position P1’ (exception (k or r,)(p)), but not when k is in position P1 and w is in position P2 at the same time (rule (w)(k,)(p)). This blocking of cleavage due to p in position P1’ is also negligible when r is in position P1 and m is in position P2 at the same time (rule (m)(r,)(p)). Furthermore, if k is found in position P1, the following situations considerably block the action of trypsin:

- Either d in position P2 and d in position P1’ (exception (d)(k,)(d))

- c in position P2 and d in position P1’ (exception (c)(k,)(d))

- c in position P2 and h in position P1’ (exception (c)(k,)(h))

- c in position P2 and y in position P1’ (exception (c)(k,)(y))

Likewise, if r is found in P1, the following situations considerably block the action of trypsin:

- Either r in position P2 and h in position P1’ (exception (r)(r,)(h))

- c in position P2 and k in position P1’ (exception (c)(r,)(k))

- r in position P2 and r in position P1’ (exception (r)(r,)(r)).

Trypsin can be defined in RPG grammar using the following rules and exceptions:

Rules:

(k,)

(r,)

(w)(k,)(p)

(m)(r,)(p)

Exceptions:

(k,)(p)

(r,)(p)

(d)(k,)(d)

(c)(k,)(d)

(c)(k,)(h)

(c)(k,)(y)

(r)(r,)(h)

(c)(r,)(k)

(r)(r,)(r)

Note that several rules and exceptions can be merged using the or keyword leading to this more compact definition:

Rules:

(k or r,)

(w)(k,)(p)

(m)(r,)(p)

Exceptions:

(k or r,)(p)

(d)(k,)(d)

(c)(k,)(d or h or y)

(r)(r,)(h or r)

(c)(r,)(k)

Other definitions of trypsin are possible using this grammar: each of these will produce the exact same result in RPG. By abstracting the programming side of new protease creation, the user can focus on the formal definition of the protease: creating new proteases is then accessible to anyone and does not require any particular programming skills.

### Comparison with existing programs

Few software exist that predict cleavage sites of proteases. Among those, the most commonly used are PeptideCutter and MaxQuant. The latter does not allow to directly digest fasta files. Moreover, while the user can design new proteases, the definition in MaxQuant only concerns the cleavage zone. Therefore, many proteases cannot be properly define.

PeptideCutter does not allow to define new proteases. The already defined proteases were thoroughly tested using RPG. All but three proteases of PeptideCutter are identically defined in RPG. The last three were defined in the local installation of RPG using the internal grammar. Despite a completely different way of expressing and defining proteases, all results of RPG are strictly identical to peptidCutter, with a better accuracy on the calculation of the molecular weight. The grammar approach of RPG can perfectly reproduce the behavior of proteases of PeptideCutter, and allows users to easily define new proteases.

### The choice of proteases

From the results in **Real case studies**, it appears that the choice of proteases is not trivial. The same combination of proteases can lead to different results depending on the nature of analyzed proteins. Here, the Actin and Globin families reveal different behaviors on two different sets of proteases. One set leads to better results for Actins, the second one for Globins. A third set leads to even better results for both families. This highlights that the digestion step of MS/MS analyses should be handled with care and should be adapted to the targeted proteins. To the best of my knowledge, there is currently no methodology nor software dedicated to this task. In the growing field of new proteases development, an ideal software should: (i) be customizable to fit user-already-acquired proteases, (ii) allow the user to employ the exact definition given by the producers, contrary to general definitions that may vary and (iii) be able to identify the optimal combination of proteases to be used for a specific set of proteins. RPG meets the first two needs.

## CONCLUSION

RPG is a new software dedicated to predict protease-induced cleavage sites. The main novelty of RPG is that it provides the user with the ability to define new proteases, hence to not be limited by RPG’s predefined proteases. To define proteases, RPG proposes an innovating yet simple grammar. This grammar uses and, or and not operators giving to the user the possibility to easily define any kind of complex proteases and yet does not require any particular programming skills. Apart from peptides, the result of a digestion includes approximations of molecular weight and isoelectric point, computed with a better accuracy than other existing software. RPG also includes two distinct digestion modes: these modes can simulate either independent digestion experiments or a single experiment of concurrent digestions by several proteases. Another advantage of RPG is that it allows the user to run all-at-once independent digestions of many proteins. Moreover, miscleavage events are taken into account in RPG. This information is of great relevance to simulate certain experimental conditions. Finally, RPG runs more than seven times faster than PeptideCutter on a single protein. Because RPG can digest multiple proteins with multiple proteases in a single run, it outperforms PeptideCutter in terms of execution time, ease of use, accuracy and functionality.

While it is beyond the scope of this article, there are two natural improvements of RPG. The first will be the creation of a dedicated website to make RPG easier to use than the current command-line program. Also, a well-conceived web interface could help the user to define new proteases. This interface could show on-the-fly the configuration of a cleaving site while rules and exceptions are inputed. A second improvement will be to automatically compute which combination of proteases leads to the best result on inputed proteins. This improvement necessitates to define what the ‘best’ result is, taking into account the number of generated peptides in a certain mass range, but also the number of the amino acids that compose those peptides. It will also require a consequent combinatorial optimization. Trying naively all possible combinations of *N* proteases requires 2^*n*^ − 1 operations: with *n* = 42 (number of available proteases by default in RPG), this leads to approximately 4.4*10^12^ operations and does not scale up. RPG was developed with adaptability in mind and these possible evolutions will not require to re-write its core.
